# Visit-to-visit fasting plasma glucose variability is associated with left ventricular adverse remodeling in diabetic patients with STEMI

**DOI:** 10.1186/s12933-020-01112-6

**Published:** 2020-09-02

**Authors:** Chen Die Yang, Ying Shen, Feng Hua Ding, Zhen Kun Yang, Jian Hu, Wei Feng Shen, Rui Yan Zhang, Lin Lu, Xiao Qun Wang

**Affiliations:** 1 Department of Cardiology, Ruijin Hospital, Shanghai Jiao-Tong University School of Medicine, 197 Ruijin Road II, Shanghai, 200025 People’s Republic of China; 2 Institute of Cardiovascular Disease, Shanghai Jiao-Tong University School of Medicine, Shanghai, People’s Republic of China

**Keywords:** FPG variability, Left ventricular adverse remodeling, Type 2 diabetes, ST-segment elevation myocardial infarction

## Abstract

**Background:**

Patients with type 2 diabetes mellitus (T2DM) are predisposed to poor cardiovascular outcomes after ST-segment elevation myocardial infarction (STEMI). Left ventricular adverse remodeling (LVAR) triggered upon myocardial infarction is recognized as the predominant pathological process in the development of heart failure. In the present study, we sought to investigate whether visit-to-visit fasting plasma glucose (FPG) variability is a potential predictor of LVAR in T2DM patients after STEMI.

**Methods:**

From January 2014 to December 2018 in Ruijin Hospital, T2DM patients with STEMI who underwent primary percutaneous coronary intervention were consecutively enrolled and followed up for ~ 12 months. The changes in left ventricular geometric and functional parameters between baseline and 12-month follow-up were assessed by echocardiography. The incidence of LVAR, defined as 20% increase in indexed left ventricular end-diastolic volume (LVEDV), and its relationship with visit-to-visit FPG variability were analyzed. Multivariate regression models were constructed to test the predictive value of FPG variability for post-infarction LVAR.

**Results:**

A total of 437 patients with type 2 diabetes and STEMI were included in the final analysis. During a mean follow-up of 12.4 ± 1.1 months, the incidence of LVAR was 20.6% and mean enlargement of indexed LVEDV was 3.31 ± 14.4 mL/m^2^, which was significantly increased in patients with higher coefficient variance (CV) of FPG (*P *= 0.002) irrespective of baseline glycemic levels. In multivariate analysis, FPG variability was independently associated with incidence of post-infarction LVAR after adjustment for traditional risk factors, baseline HbA1c as well as mean FPG during follow-up (OR: 3.021 [95% CI 1.081–8.764] for highest vs. lowest tertile of CV of FPG). Assessing FPG variability by other two measures, including standard deviation (SD) and variability independent of the mean (VIM), yielded similar findings.

**Conclusions:**

This study suggests that visit-to-visit FPG variability is an independent predictor of incidence of LVAR in T2DM patients with STEMI.

*Trial registration* Trials number, NCT02089360; registered on March 17,2014.

## Background

Due to rapid advances in percutaneous coronary intervention (PCI) technique and guideline-based medical therapy, the early and late mortality in ST-segment elevation myocardial infarction (STEMI) patients has considerably declined over the past decades [1]. However, recurrence of ischemic events and progression of heart failure (HF) evidently affect post-infarction survival especially in patients with comorbidities or with inappropriate management measures [2–4]. Upon myocardial infarction (MI), left ventricular adverse remodeling (LVAR) is triggered in response to abrupt increase in wall stress and distension in the infarct area, which is recognized as the central pathological process in the development of HF after MI [5, 6].

Type 2 diabetes mellitus (T2DM) is a well-established risk factor for post-infarction HF and mortality [7–10]. Compared with non-diabetic patients, diabetic patients generally exhibit similar LVAR but higher left ventricular (LV) filling pressure following MI, which to some extent contributes to the pathogenesis of post-infarction HF [3, 11, 12]. Hyperglycemia is one of the key factors in the development of post-infarction LVAR, partly by promoting remodeling-related gene expression, cardiomyocyte apoptosis and interstitial fibrosis [13–15]. A recent retrospective study demonstrated a J-shaped association between basal fasting glucose levels in the acute phase and risk of mortality (*P *= 0.0001), but a direct association with HF (*P* = 0.03) [16, 17]. Basal hyperglycemia was also showed to be independently correlated with LVAR at 6 months after STEMI (*P *< 0.001) [18].

On the other hand, two recent studies showed that in STEMI patients underwent primary PCI, greater in-hospital fasting plasma glucose (FPG) variability determined by a continuous glucose monitoring system (CGMS) was associated with not only poor short-term prognosis but also the development of LVAR in chronic phase [19, 20]. The detrimental influence of FPG variability on LV enlargement in addition to classical glucose metrics was also confirmed in the context of experimental myocardial ischemia/reperfusion [21]. However, the relationship between long-term FPG variability and post-infarction LVAR is still unclear. Therefore, in the present study, we sought to investigate whether visit-to-visit FPG variability predicts LVAR in patients with type 2 diabetes after STEMI with primary PCI.

## Methods

### Study population

**Fig. 1:**
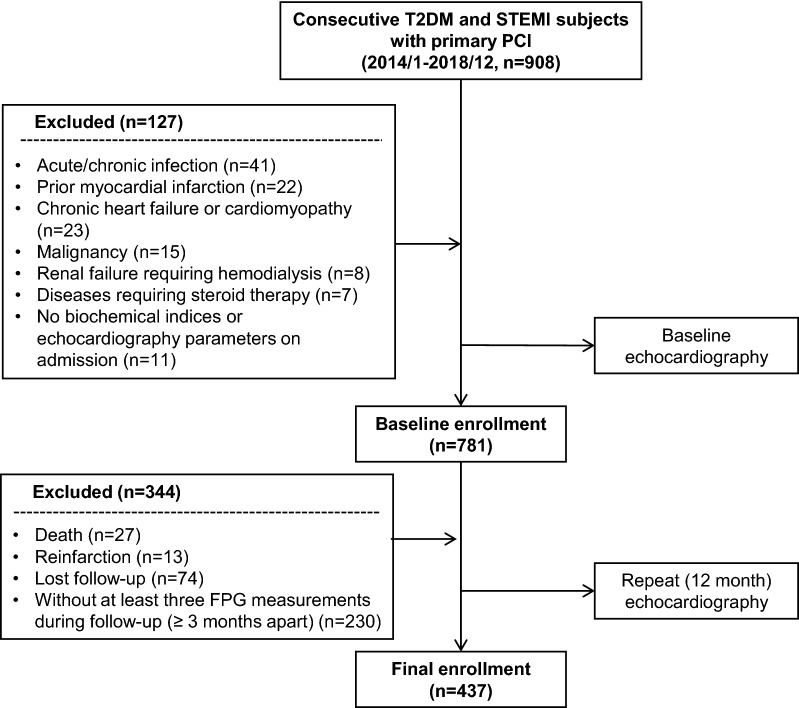
Flow chart of patient enrollment. *FPG* fasting plasma glucose, *STEMI* ST-segment elevation myocardial infarction, *T2DM* type 2 diabetes mellitus, *PCI* percutaneous coronary intervention

We consecutively enrolled 908 T2DM subjects with acute STEMI within 12 h of onset of symptoms and received primary PCI from Jan, 2014 to Dec, 2018 in the Department of Cardiology, Ruijin Hospital, Shanghai Jiao Tong University School of Medicine. A total of 127 patients comorbid with chronic or acute infection, prior MI, chronic HF or cardiomyopathy, malignancy, renal failure requiring hemodialysis, diseases requiring steroid therapy, as well as those with no biochemical indices or echocardiography parameters before discharge were excluded. During follow-up, there were 27 death, 13 reinfarction and 74 lost to follow-up, which were also excluded. For calculation of FPG variability, subjects (n = 230) without at least three FPG measurements during follow-up (≥ 3 months apart) were further excluded. Thus, 437 patients underwent follow-up echocardiography at around 12-month and comprised the final enrollment (Fig. 1).

This study complies with the Declaration of Helsinki. The study protocol was approved by the local hospital ethics committee, and written informed consent was obtained from all participants.

### Clinical, biochemical and echocardiographic assessments

The detailed information about medical history and lifestyles including smoking habits was obtained using a standard questionnaire by trained physicians. Body mass index (BMI) was calculated as weight/height^2^ (kilograms per square meter). Body surface area (BSA) was calculated as 0.0061 × height + 0.0128 × weight − 0.1529. Blood pressure was measured on the non-dominant arm in seated position after a 10-min rest. Three measurements were taken at 1-min interval, and the average was used for analysis. Hypertension was diagnosed according to seventh report of the Joint National Committee on prevention, detection, evaluation, and treatment of high blood pressure (JNC 7) [22]. The diagnosis of T2DM was made according to the criteria of American Diabetes Association [23].

All the blood samples were drawn after an overnight fasting, except for peak troponin I which was the highest level of serial measurements during hospitalization. Plasma glucose, serum insulin, creatinine, total cholesterol, low-density lipoprotein (LDL) cholesterol, high-density lipoprotein (HDL) cholesterol, triglycerides were assessed (HITACHI 912 Analyzer, Roche Diagnostics, Germany). The estimated glomerular filtration rate (eGFR) was computed using the Chronic Kidney Disease Epidemiology Collaboration equation [24]. Blood HbA1c was measured using ion-exchange high performance liquid chromatography with Bio-rad Variant Hemoglobin Testing System (Bio-Rad Laboratories, USA). Serum levels of high sensitive C-reactive protein (hsCRP) were determined by ELISA (Biocheck Laboratories, Toledo, OH, USA).

Transthoracic echocardiography was performed, at least, at the time of enrollment and 12-month follow-up, using a commercially available system (Vivid-I, GE Healthcare, Milwaukee, WI) with a 1.9- to 3.8-mHz phased-array transducer. Two-dimensional (2D), pulsed-Doppler imaging was performed from standard parasternal and apical transducer positions with 2D frame rates of 60 to 100 frames/s. All data were stored digitally, and offline data analysis was performed (EchoPac, version 7; GE Healthcare) by two cardiologists at the conclusion of the study, blinded to the study time point.

The LV ejection fraction (LVEF) was calculated using the modified Simpson’s biplane technique. The LV length was measured in the apical 4-chamber view. To facilitate application of clinical normality cut points, LV end-diastolic volume (LVEDV) and LV end-systolic volume (LVESV) and LV mass were indexed by BSA calculated at each study time point. LV mass was estimated from M-mode measurements by the formula:

$${\text{LV mass}}\, = \,0.8 \times 1.04 \times \left[ {\left( {LVEDD + IVST + LVPWT} \right)^{3} - LVEDD^{3} } \right] + 0.6,$$

where LVEDD is LV end-diastolic diameter, IVST is interventricular septal thickness, LVPWT is LV posterior wall thickness. Relative wall thickness (RWT) was determined by the formula:

$$RWT = \left( {IVST + LVPWT} \right)/LVEDD$$

### FPG variability determinations

Visit-to-visit FPG was measured during follow-up period for at least three times in 3-month intervals. Then the mean and variability of FPG were calculated. FPG variability was primarily defined as intraindividual coefficient of variation (CV) of FPG across visits. The alternative variability of FPG includes: 1) standard deviation (SD) and 2) the variability independent of the mean (VIM), which was calculated by the equation as previously reported [25]: VIM = 100 × SD/mean^β^, where β is the regression coefficient based on natural logarithm of SD on natural logarithm of mean of the study population.

### Statistical analysis

Continuous variables were presented as median (interquartile range [IQR]) or mean ± SD, and categorical data were summarized as frequencies (percentages). Normal distribution of continuous variables was evaluated by Shapiro–Wilk test. For normally distributed variables, differences in tertiles of FPG variability and subgroup analysis were performed by one-way or two-way analysis of variance (ANOVA) followed by post hoc *t* test with Bonferroni correction. For non-normally distributed continuous variables, differences were analyzed by Mann–Whitney U test or Kruskal–Wallis test. Differences in categorical variables were analyzed by χ^2^ test. Multivariate regression models were constructed to interrogate the association between FPG variability and LVAR, which is generally defined as 20% increase in indexed LVEDV [5]. In model 1, sex and age were adjusted; In model 2, additional adjustment was performed for history of hypertension, duration of diabetes, smoking status, baseline HbA1c, postprandial plasma glucose, non-HDL cholesterol, eGFR, the presence of multivessel disease (MVD), peak value of troponin I and baseline LVEF; In model 3, we further adjusted for medication use of oral hypoglycemic agents (OHA), insulin, beta blocker and angiotensin-converting enzyme inhibitor (ACEI)/angiotensin receptor blocker (ARB). In model 4, mean level of FPG during follow-up was additionally adjusted.

All statistical analyses were performed using the R statistical package v.3.6.3 (R Project for Statistical Computing, Vienna, Austria). A 2-tailed < 0.05 was considered statistically significant.

## Results

### Basic characteristics of the study population

A total of 437 T2DM patients with STEMI who underwent primary PCI were included in the final analysis. The mean age was 63.0 ± 11.5 years with 84.7% male patients. Among these subjects, 62.2% were with hypertension and 69.6% were with MVD. The mean number of intrapersonal FPG tests was 4.46 ± 1.57 and the mean FPG level during follow-up was 7.22 ± 2.13 mmol/L, and CV, SD, VIM of FPG during follow-up were 0.214 [IQR 0.111–0.334], 1.470 [IQR 0.671–2.740] and 1.060 [IQR 0.668–1.550], respectively. To analyze the effect of FPG variability on post-infarction LVAR, we divided the population based on tertiles of CV of visit-to-visit FPG (Table 1). There was no significant difference in age, sex, BMI, baseline blood pressure, smoking habits, HDL cholesterol, renal function and hsCRP between the three tertiles. On admission, subjects with the highest tertile of CV of FPG had longer duration of diabetes, higher levels of HbA1c, FPG and fasting insulin level, postprandial plasma glucose, LDL cholesterol and peak values of troponin I, but lower postprandial insulin level than those with the lowest tertile. In addition, patients with the highest tertile were with more severely diseased vessels and more frequently used insulin.

**Table 1:** Baseline characteristics

Tertiles of CV of FPG	T1 ≤ 0.157	T2 0.157–0.249	T3 > 0.249	*P* value
n	146	146	145	
Demographic characteristics & clinical measures				
Male	126 (86.3)	124 (84.9)	120 (82.8)	0.702
Age, years	61.79 ± 11.24	63.15 ± 11.54	63.96 ± 11.67	0.268
BMI, kg/m^2^	24.68 ± 3.12	25.18 ± 4.78	25.28 ± 3.48	0.387
Systolic BP, mmHg	122.26 ± 17.43	124.47 ± 19.92	120.75 ± 20.56	0.284
Diastolic BP, mmHg	82.78 ± 81.65	75.56 ± 12.19	72.83 ± 12.42	0.217
Medical history				
Hypertension	76 (52.1)	104 (71.2)	92 (63.4)	0.003
Duration of diabetes, years	6.38 ± 3.93	6.68 ± 3.90	10.77 ± 7.16	< 0.001
Current smoker	68 (46.6)	76 (52.1)	65 (44.8)	0.456
Laboratory values				
HbA1_C_, %	6.23 ± 0.94	6.69 ± 1.41	7.54 ± 1.70	< 0.001
FPG, mmol/L	5.77 (5.12–6.70)	7.67 (6.76–9.33)	10.70 (7.91–13.34)	< 0.001
PPG (2 h), mmol/L	8.65 (6.88–11.27)	11.18 (7.59–12.97)	11.79 (9.71–15.56)	< 0.001
Fasting insulin, µU/mL	10.72 (8.35–15.52)	9.42 (5.80–15.80)	12.00 (8.33–24.78)	0.003
Postparandial insulin (2 h), µU/mL	56.23 (33.60–109.03)	37.31 (16.96–65.80)	27.92 (13.27–75.63)	< 0.001
Triglyceride, mmol/L	1.63 (1.32–2.21)	1.54 (1.02–2.05)	1.58 (1.11–2.02)	0.033
Total cholesterol, mmol/L	4.36 ± 0.95	4.53 ± 1.18	4.56 ± 1.35	0.273
HDL cholesterol, mmol/L	0.99 ± 0.28	1.02 ± 0.27	1.01 ± 0.24	0.734
LDL cholesterol, mmol/L	2.57 ± 0.86	2.81 ± 0.95	2.89 ± 1.15	0.020
Non-HDL cholesterol,	3.36 ± 0.96	3.51 ± 1.12	3.53 ± 1.32	0.388
eGFR, mL/min/1.73m2	93.90 ± 15.42	97.26 ± 22.73	97.93 ± 33.81	0.343
hsCRP, mg/L	3.01 (1.28–8.28)	2.73 (0.84–19.00)	4.88 (1.79–26.90)	0.062
Peak troponin I	4.23 (0.40–15.85)	8.37 (3.34–48.53)	8.80 (4.63–69.11)	< 0.001
Diseased vessels				
1-vessel	50 (34.2)	50 (34.2)	33 (22.8)	
2-vessel	52 (35.6)	64 (43.8)	61 (42.1)	0.040
3-vessel	44 (30.1)	32 (21.9)	51 (35.2)	
MVD	96 (65.8)	96 (65.8)	112 (77.2)	0.056
Culprit vessel				
LM	0 (0.0)	2 (1.3)	4 (2.3)	
LAD	86 (55.1)	98 (62.0)	86 (50.0)	
LCX	28 (17.9)	20 (12.7)	39 (22.7)	0.100
RCA	42 (26.9)	38 (24.1)	43 (25.0)	
Medication use, n(%)				
Aspirin	138 (94.5)	138 (94.5)	133 (91.7)	0.546
P2Y12 inhibitor	138 (94.5)	134 (91.8)	130 (89.7)	0.311
Beta blocker	134 (91.8)	122 (83.6)	122 (84.1)	0.064
ACEI/ARB	112 (76.7)	110 (75.3)	105 (72.4)	0.694
CCB	6 (4.1)	14 (9.6)	25 (17.2)	0.001
Statin	104 (71.2)	120 (82.2)	132 (91.0)	< 0.001
Biguanides	14 (9.6)	20 (13.7)	38 (26.2)	< 0.001
Sulfonylureas	6 (4.1)	16 (11.0)	24 (16.6)	0.002
Meglitinides	4 (2.7)	0 (0.0)	12 (8.3)	0.002
Glucosidase inhibitor	80 (54.8)	66 (45.2)	60 (41.4)	0.064
SGLT2 inhibitor	2 (1.4)	0 (0.0)	1 (0.7)	0.548
OHA	92 (63.0)	84 (57.5)	86 (59.3)	0.644
Insulin	4 (2.7)	8 (5.5)	26 (17.9)	< 0.001

*ACEI* angiotensin-converting enzyme inhibitor, *ARB* angiotensin receptor blocker, *BMI* body mass index, *BP* blood pressure, *CCB* calcium channel blockers, *CV* coefficient of variation, *eGFR* estimated glomerular filtration rate, *FPG* fasting plasma glucose, *HbA1c* glycated hemoglobin A1c, *HDL* high-density lipoprotein, *hsCRP* high-sensitivity C-reactive protein, *LAD* left anterior descending branch, *LCX* left circumflex branch, *LDL* low-density lipoprotein, *LM* left main coronary artery, *MVD* multi-vessel disease, *OHA* oral hypoglycemic agent, *PPG* postprandial plasma glucose, *RCA* right coronary artery, *SGLT2* sodium-dependent glucose transporters 2

### Changes in LV geometric and functional properties

Baseline and 12-month follow-up LV geometric and functional parameters were assessed. In the overall population, the mean changes of LVEDD and indexed LVEDV were 1.01 ± 4.20 mm and 3.31 ± 14.4 mL/m^2^. The incidence of LVAR, generally defined as 20% increase in indexed LVEDV, was 20.6%. Compared with subjects with optimized glycemic control (mean FPG < 6.8 mmol/L), those with suboptimal glycemic control (mean FPG ≥ 6.8 mmol/L) presented greater LV enlargement during follow-up (5.41 ± 15.3 vs. 1.15 ± 1.32 mL/m^2^, *P *= 0.003).

**Fig. 2:**
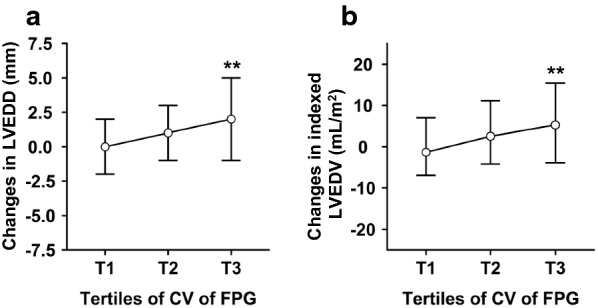
Distribution of changes in LV dimension among tertiles of CV of FPG. Shown are distribution of changes in LVEDD (**a**) and indexed LVEDV (**b**) according to tertiles of CV of FPG. Data are expressed as median (IQR). **P < 0.01 vs. subjects with lowest tertile of CV of FPG. *CV* coefficient of variance, *FPG* fasting plasma glucose, *IQR* interquartile range, *LV* left ventricular, *LVEDD* left ventricular end-diastolic diameter, *LVEDV* left ventricular end-diastolic volume

Changes in echocardiography parameters were compared in subjects stratified by tertiles of CV of visit-to-visit FPG (Table 2). There was an upward trend in post-infarction LV enlargement with increasing tertiles (Fig. 2; Δ LVEDD, *P *< 0.001; Δ indexed LVEDV, *P* = 0.002). Increase in indexed LV mass (*P *= 0.050) and decrease in RWT (*P *= 0.020) were borderline significant in patients with higher CV of FPG during follow-up. Recovery of cardiac function during follow-up was more prominent in those with the lowest tertile (*P* = 0.044).

**Table 2:** Changes in echocardiography parameters during follow-up grouped by tertiles of CV of FPG

Tertiles of CV of FPG		T1	T2	T3	*P* value
		≤ 0.157	0.157–0.249	> 0.249	
n		146	146	145	
LVEDD (mm)	B	51.48 ± 3.89	51.62 ± 4.90	50.21 ± 4.67	0.014
	F	51.49 ± 4.59	52.60 ± 5.26	52.24 ± 5.43	0.167
	Δ	0.01 ± 3.83	0.99 ± 3.81	2.03 ± 4.69	< 0.001
LVESD (mm)	B	35.82 ± 4.98	36.00 ± 5.33	35.51 ± 5.00	0.710
	F	34.99 ± 5.83	36.51 ± 6.13	36.29 ± 6.58	0.077
	Δ	− 0.84 ± 4.41	0.51 ± 3.83	0.78 ± 5.01	0.004
Indexed LVEDV (mL/m2)	B	72.19 ± 13.89	72.32 ± 16.44	68.41 ± 13.70	0.048
	F	72.83 ± 16.31	74.94 ± 16.55	75.14 ± 18.57	0.471
	Δ	0.64 ± 13.01	2.62 ± 12.93	6.73 ± 16.51	0.002
Indexed LVESV (mL/m2)	B	31.90 ± 11.84	31.95 ± 12.05	31.17 ± 10.22	0.819
	F	30.31 ± 12.95	32.77 ± 13.70	33.17 ± 15.96	0.204
	Δ	− 1.59 ± 9.82	0.82 ± 8.73	2.00 ± 12.71	0.018
IVSI (mm)	B	9.52 ± 1.14	9.38 ± 1.54	9.39 ± 1.20	0.606
	F	9.34 ± 1.28	9.15 ± 1.16	9.26 ± 1.15	0.391
	Δ	− 0.18 ± 1.46	− 0.23 ± 1.50	− 0.14 ± 1.38	0.854
LVPWI (mm)	B	9.07 ± 0.84	8.89 ± 0.82	9.17 ± 1.11	0.034
	F	8.92 ± 0.87	8.75 ± 0.89	8.85 ± 0.77	0.251
	Δ	− 0.15 ± 1.27	− 0.14 ± 1.12	− 0.32 ± 1.13	0.319
RWT (mm)	B	0.36 ± 0.04	0.36 ± 0.05	0.37 ± 0.05	0.016
	F	0.36 ± 0.05	0.34 ± 0.04	0.35 ± 0.05	0.024
	Δ	− 0.00 ± 0.06	− 0.01 ± 0.05	− 0.02 ± 0.05	0.020
Indexed LV Mass (g/m2)	B	97.69 ± 18.66	96.05 ± 21.54	92.91 ± 19.14	0.132
	F	95.77 ± 19.01	95.54 ± 21.94	96.63 ± 20.46	0.901
	Δ	− 1.92 ± 19.61	− 0.50 ± 17.76	3.72 ± 22.58	0.057
LVEF (%)	B	56.74 ± 8.93	56.40 ± 8.05	55.26 ± 8.00	0.284
	F	59.71 ± 8.38	57.25 ± 8.18	57.23 ± 9.14	0.018
	Δ	2.97 ± 8.09	0.85 ± 6.52	1.97 ± 7.02	0.044

*B* baseline, *Δ* changes in corresponding parameters, *F* follow-up, *CV* coefficient of variance, *FPG* fasting plasma glucose, *IVST* interventricular septal thickness, *LV* left ventricular, *LVEDD* left ventricular end-diastolic diameter, *LVEDV* left ventricular end-diastolic volume, *LVEF* left ventricular ejection fraction, *LVESD* left ventricular end-systolic diameter, *LVESV* left ventricular end-systolic volume, *LVPWT* left ventricular posterior wall thickness, *RWT* relative wall thickness

**Fig. 3:**
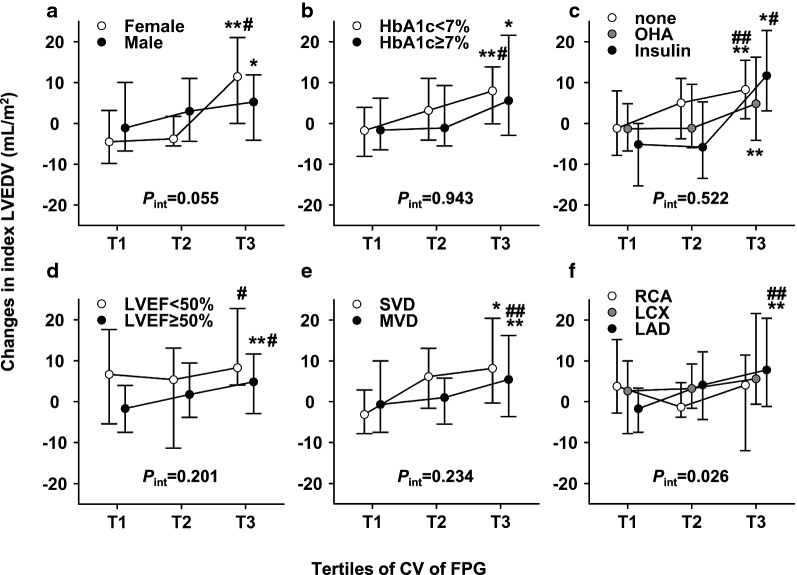
The impact of FPG variability on changes in LV dimension across subgroups. The impact of FPG variability on changes in indexed LVEDV was analyzed across subgroups of sex (**a**), baseline HbA1c (**b**), hypoglycemic therapy (**c**), baseline LVEF (**d**), the presence of multi-vessel disease (**e**) and the culprit vessel (**f**). *P < 0.05 vs. subjects with lowest tertile of CV of FPG; **P < 0.01 vs. subjects with lowest tertile of CV of FPG. ^#^*P *< 0.05 vs. subjects with intermediate tertile of CV of FPG; ^##^*P *< 0.01 vs. subjects with intermediate tertile of CV of FPG. *CV* coefficient of variance, *FPG* fasting plasma glucose, *HbA1c* glycated hemoglobin A1c, *LAD* left anterior descending branch, *LCX* left circumflex branch, *LV* left ventricular, *LVEDV* left ventricular end-diastolic volume, *LVEF* left ventricular ejection fraction, *MVD* multi-vessel disease, *OHA* oral hypoglycemia agent, *RCA* right coronary artery, *SVD* single-vessel disease

Subgroup analyses were then performed to investigate the impact of FPG variability on post-infarction LV remodeling (Fig. 3). Consistent LV enlargement was found in patients with higher CV of FPG irrespective of sex, baseline HbA1c, hypoglycemic therapy, baseline LVEF or the presence of MVD. Infarction of left anterior descending branch (LAD) dominant segments but not of other vessels exhibited greater LV enlargement across the tertiles. There was no significant interaction term between tertiles of CV of FPG and each of these grouping variables, with the solo exception for the culprit vessel (*P* = 0.026).

### Multivariate analysis

Multivariate analysis was performed to analyze the association between the incidence of LVAR and different measures of FPG variability (Table 3). The age- and sex- adjusted OR for LVAR in subjects with the highest tertile versus the lowest tertile was 1.879 [95% CI 1.067–3.364]. After multivariate adjustment (model 3), the highest tertile conferred a higher risk of LVAR as compared to the lowest tertile (3.333 [95% CI 1.301–8.953]). After additional adjustment for mean FPG during follow-up (model 4), the corresponding OR for LVAR in the highest tertile versus the lowest tertile remained significant (3.021 [95% CI 1.081–8.764]). Similar findings were observed by inclusion of other measures of FPG variability into these models instead. After stratifying the population based on mean FPG level during follow-up, VIM remained associated with LVAR in patients with suboptimal glucose control (≥ 6.8 mmol/L) but not in those with optimal glucose control (< 6.8 mmol/L) in the full adjustment model (model 3 in Additional file 1: Table S1).

**Table 3:** Multivariate regression analysis for LVAR after STEMI

	Model 1		Model 2		Model 3		Model 4	
	OR (95% CI)	*P*	OR (95% CI)	*P*	OR (95% CI)	*P*	OR (95% CI)	*P*
CV		0.026*		0.031*		0.015*		0.035*
T1	Reference	–	Reference	–	Reference	–	Reference	–
T2	1.080 (0.586–2.000)	0.804	1.048 (0.444–2.496)	0.915	1.116 (0.457–2.757)	0.810	1.049 (0.412–2.682)	0.920
T3	1.879 (1.067–3.364)	0.031	2.652 (1.110–6.551)	0.030	3.333 (1.301–8.953)	0.014	3.021 (1.081–8.764)	0.037
SD		0.005*		0.012*		0.005*		0.013*
T1	Reference	–	Reference	–	Reference	–	Reference	–
T2	1.202 (0.645–2.255)	0.563	0.835 (0.341–2.032)	0.689	0.846 (0.336–2.125)	0.721	0.830 (0.313–2.184)	0.705
T3	2.265 (1.279–4.100)	0.006	3.063 (1.277–7.613)	0.013	3.963 (1.541–10.667)	0.005	3.832 (1.307–11.658)	0.016
VIM		0.364*		0.060*		0.017*		0.012*
T1	Reference	–	Reference	–	Reference	–	Reference	–
T2	1.664 (0.935–3.002)	0.086	2.877 (1.289–6.860)	0.012	3.499 (1.500–8.736)	0.005	3.314 (1.403–8.344)	0.008
T3	1.333 (0.735–2.438)	0.345	2.379 (0.998–5.932)	0.055	3.196 (1.273–8.475)	0.016	3.404 (1.342–9.142)	0.012

Model 1, includes adjustment for age and sex

Model 2, additional adjustment for history of hypertension, duration of diabetes, smoking status, baseline HbA1c, postprandial plasma glucose, non-HDL cholesterol, eGFR, the presence of multivessel disease, peak value of troponin I and baseline LVEF

Model 3, additional adjustment for medication use of oral hypoglycemic agents, insulin, beta blocker and ACEI/ARB

Model 4, additional adjustment for mean FPG level during follow-up

* *P* for trend. *ACEI* angiotensin-converting enzyme inhibitor; *ARB* angiotensin receptor blocker, *CI* confidence interval, *CV* coefficient of variation, *eGFR* estimated glomerular filtration rate, *FPG* fasting plasma glucose, *HbA1c* glycated hemoglobin A1c, *HDL* high-density lipoprotein, *LVAR* left ventricular adverse remodeling, *LVEF* left ventricular ejection fraction, *OR* odds ratio, *SD* standard deviation, *STEMI* ST-segment elevation myocardial infarction, *VIM* variability independent of the mean

## Discussion

The major findings of the present study are that in T2DM patients with STEMI, LV enlargement is more prominent in those with high visit-to-visit FPG variability. FPG variability is an independent predictor for the development of LVAR even after adjustment for mean glycemic control levels.

### FPG variability predicts LVAR after STEMI

LVAR after MI is generally considered to be associated with the incidence of HF and poor cardiovascular outcomes [5, 6]. Previous reports showed that diabetic patients in general had a similar incidence of post-infarction LVAR as non-diabetic patients, but with significantly higher risk of HF and cardiovascular mortality [2, 11]. Multiple mechanisms are implicated in the process of LVAR including chronic hyperglycemia. A retrospective study analyzing 52 patients with STEMI showed that basal hyperglycemia (glucose levels were above 123.5 mg/dL [6.86 mmol/L]) was independently correlated with LV enlargement at 6 months after STEMI (*P* < 0.001) [18]. Another basic research revealed that hyperglycemia exaggerated LV remodeling and failure after MI by increasing interstitial fibrosis and myocyte apoptosis [14]. Consistent with these findings, our study showed that T2DM patients with suboptimal glycemic control (mean FPG ≥ 6.8 mmol/L) tended to have greater LV enlargement than those with optimal glycemic control (mean FPG < 6.8 mmol/L).

Being regarded as another key component in glucose homeostasis, FPG variability confers unfavorable impacts on the development of diabetic micro- and macrovascular complications, myocardial diastolic function, cardiovascular risk, prognosis of acute disease and all-cause mortality in diabetic patients [26–30]. Especially, short-term FPG variability determined by CGMS was shown to predict LVAR during a 7-month follow-up in patients with STEMI [20]. However, whether long-term FPG variability affects LVAR in chronic phase in diabetic patients after STEMI is still unclear to our knowledge. A recent study showed that visit-to-visit variability in FPG is a risk factor for the long-term adverse changes in left cardiac structure and systolic function in general T2DM patients [31]. An animal study using cardiac magnetic resonance showed that FPG variability induced by intermittent insulin injection during the peri-procedural period led to adverse ventricular enlargement after experimental myocardial ischemia/reperfusion injury [21]. In fact, given maladaptive myocardial remodeling is a long-term process, it is rationale to evaluate the impact of glycemic level and stability on LVAR in the long term, on which our study is based.

In the present study, we for the first time reported that in diabetic patients with STEMI, patients with high visit-to-visit FPG variability tended to have greater LV enlargement during follow-up. Subgroup analysis showed such trend persisted irrespective of glycemic level. Furthermore, multivariate analyses demonstrated that long-term FPG variability was independently associated with post-infarction LVAR even after adjustment for baseline HbA1c as well as mean FPG during follow-up. In addition, we assessed FPG variability by different measures including SD, CV and VIM. CV is relatively simple and more feasible in clinical practice, whereas VIM is calculated based on logarithmic curve fitting to eliminate its correlation with mean FPG. We showed that all these measures of FPG variability yielded similar findings. Taken together, our study supports the notion that long-term FPG variability is critical in the process of LVAR after MI in T2DM patients. Variability of FPG adds to its mean level for risk prediction of LVAR. Indeed, a previous study of experimental myocardial ischaemia/reperfusion also showed that FPG variability posed a more deleterious effect on myocardium than permanently high blood glucose levels [21].

Noteworthy, previous studies suggest comparable or less LV enlargement in diabetic patients than non-diabetic subjects after MI [2, 3, 11]. In line with previous findings, we showed a moderate increase in indexed LVEDV (follow-up: 74.30 ± 17.16 vs. 70.99 ± 14.81 mL/m^2^, *P *< 0.001), and non-significant change in indexed LVESV (follow-up: 32.08 ± 14.27 vs. 31.68 ± 11.39 mL/m^2^, *P *= 0.448) in the overall population of the cohort. Importantly, our findings imply that the pattern of post-infarction LV remodeling may differ according to glycemic control and variability in diabetic patients. Patients with high FPG variability presented greater LV enlargement and perceived a 3.021-fold increase in the incidence of post-infarction LVAR compared to those with low FPG variability, suggesting a trend towards eccentric remodeling in diabetic patients with unstable glycemic control.

### Possible mechanisms

The mechanisms by which FPG variability affects the development of LVAR after MI remain poorly understood. Previous basic studies showed in the setting of MI, glycemic fluctuation promotes the production of reactive oxygen species, mitochondrial stress, coronary microvascular dysfunction and impaired ischaemia-induced angiogenesis[32–37]. These factors potentially lead to limited myocardial blood reperfusion, compromised myocardial salvage, persistent myocardial ischemia, maladaptive myocardial remodeling, and finally the development of HF. Nevertheless, the specific mechanisms await precise characterization in future studies and are our important future goal.

### Study limitation

Our findings should be interpreted in the context of following limitations. First, this study is a retrospective analysis based on prospectively collected data, and all the enrolled patients were from a single center. Second, LV diastolic function such as E/A ratio was not assessed, which was previously reported to be a key player in LV remodeling after MI among diabetic patients. Third, we evaluated LV geometry and function by echocardiography rather than cardiac magnetic resonance, which may provide more precise information. Fourth, FPG variability in the study was evaluated based on sequential FPG measurements during follow-up. HbA1c variability might reflect different aspects of FPG variability and deserves further study. Finally, prospective studies are warranted to analyze the causal link between glycemic variability and post-infarction LVAR, as well as the prognostic value of long-term glycemic variability for hard cardiovascular events in T2DM subjects with STEMI.

## Conclusions

In conclusion, our findings suggest that greater visit-to-visit FPG variability is associated with higher incidence of LVAR in T2DM patients with STEMI. Variability of FPG adds to its mean level for risk prediction of post-infarction LVAR.

## Supplementary information

**Additional file 1: Table S1.** Multivariate regression analysis for LVAR after STEMI stratified by mean FPG level.

## Data Availability

The datasets used and/or analyzed during the current study are available from the corresponding author on reasonable request.

## Abbreviations

ACEI: Angiotensin-converting enzyme inhibitor
ANOVA: Analysis of variance
ARB: Angiotensin receptor blocker
BMI: Body mass index
BSA: Body surface area
CGMS: Continuous glucose monitoring system
CV: Coefficient of variance
eGFR: Estimated glomerular filtration rate
FPG: Fasting plasma glucose
HDL: High-density lipoprotein
HF: Heart failure
hsCRP: High sensitive C-reactive protein
IQR: Interquartile range
IVST: Interventricular septal thickness
LAD: Left anterior descending branch
LDL: Low-density lipoprotein
LV: Left ventricular
LVAR: Left ventricular adverse remodeling
LVEDD: Left ventricular end-diastolic diameter
LVEDV: Left ventricular end-diastolic volume
LVEF: Left ventricular ejection fraction
LVESV: Left ventricular end-systolic volume
LVPWT: left ventricular posterior wall thickness
MI: Myocardial infarction
MVD: Multivessel disease
OHA: Oral hypoglycemic agent
PCI: Percutaneous coronary intervention
RWT: Relative wall thickness
SD: Standard deviation
STEMI: ST-segment elevation myocardial infarction
T2DM: Type 2 diabetes mellitus
VIM: Variability independent of the mean
