# Supplementary material for: Visit-to-visit fasting plasma glucose variability is associated with left ventricular adverse remodeling in diabetic patients with STEMI
Source: Cardiovasc Diabetol. 2020 Sep 2;19:131. doi: 10.1186/s12933-020-01112-6 (PMC7469406; doi:10.1186/s12933-020-01112-6)
Supplement: Supplementary file 1 — Additional file 1: Table S1. Multivariate regression analysis for LVAR after STEMI stratified by mean FPG level. [file 12933_2020_1112_MOESM1_ESM.docx]

|  |  |  | **Model 1** | |  | **Model 2** | |  | **Model 3** | |
| --- | --- | --- | --- | --- | --- | --- | --- | --- | --- | --- |
|  |  |  | **OR (95% CI)** | ***P*** |  | **OR (95% CI)** | ***P*** |  | **OR (95% CI)** | ***P*** |
| **Mean FPG <6.8 mmol/L** | **CV** |  |  | 0.943* |  |  | 0.540* |  |  | 0.845* |
|  |  | **T1** | Reference | *-* |  | Reference | *-* |  | Reference | *-* |
|  |  | **T2** | 1.021 (0.391~2.672) | 0.965 |  | 1.238 (0.421~3.695) | 0.697 |  | 1.944 (0.595~6.755) | 0.278 |
|  |  | **T3** | 1.035 (0.398~2.692) | 0.943 |  | 0.646 (0.182~2.160) | 0.482 |  | 0.786 (0.209~2.880) | 0.715 |
|  | **SD** |  |  | 0.681* |  |  | 0.377* |  |  | 0.565* |
|  |  | **T1** | Reference | *-* |  | Reference | *-* |  | Reference | *-* |
|  |  | **T2** | 1.278 (0.509~3.272) | 0.602 |  | 1.473 (0.523~4.242) | 0.464 |  | 2.565 (0.804~8.907) | 0.121 |
|  |  | **T3** | 0.800 (0.288~2.158) | 0.660 |  | 0.476 (0.112~1.739) | 0.279 |  | 0.507 (0.111~2.034) | 0.351 |
|  | **VIM** |  |  | 0.569* |  |  | 0.807* |  |  | 0.871* |
|  |  | **T1** | Reference | *-* |  | Reference | *-* |  | Reference | *-* |
|  |  | **T2** | 0.804 (0.289~2.170) | 0.667 |  | 0.776 (0.254~2.322) | 0.65 |  | 0.788 (0.246~2.457) | 0.682 |
|  |  | **T3** | 1.298 (0.519~3.310) | 0.577 |  | 0.884 (0.277~2.761) | 0.832 |  | 0.932 (0.282~3.089) | 0.907 |
| **Mean FPG ≥6.8 mmol/L** | **CV** |  |  | 0.685* |  |  | 0.951* |  |  | 0.408* |
|  |  | **T1** | Reference | - |  | Reference | - |  | Reference | - |
|  |  | **T2** | 1.647 (0.787~3.494) | 0.187 |  | 2.076 (0.883~5.032) | 0.098 |  | 2.994 (1.059~9.090) | 0.044 |
|  |  | **T3** | 0.847 (0.384~1.856) | 0.679 |  | 1.054 (0.380~2.914) | 0.919 |  | 1.695 (0.484~6.092) | 0.410 |
|  | **SD** |  |  | 0.688* |  |  | 0.931* |  |  | 0.426* |
|  |  | **T1** | Reference | - |  | Reference | - |  | Reference | - |
|  |  | **T2** | 1.649 (0.788~3.499) | 0.187 |  | 1.994 (0.840~4.861) | 0.121 |  | 2.783 (0.994~8.352) | 0.057 |
|  |  | **T3** | 0.848 (0.384~1.857) | 0.680 |  | 0.929 (0.290~2.904) | 0.899 |  | 1.467 (0.331~6.617) | 0.613 |
|  | **VIM** |  |  | 0.212* |  |  | 0.093* |  |  | 0.003* |
|  |  | **T1** | Reference | - |  | Reference | - |  | Reference | - |
|  |  | **T2** | 2.493 (1.160~5.563) | 0.022 |  | 4.817 (1.920~13.323) | 0.001 |  | 9.113 (2.967~32.619) | <0.001 |
|  |  | **T3** | 1.707 (0.767~3.899) | 0.194 |  | 2.563 (0.917~7.652) | 0.079 |  | 7.001 (2.006~28.016) | 0.003 |

**Additional file Table S1. Multivariate regression analysis for LVAR after STEMI stratified by mean FPG level**

Model 1, includes adjustment for age and sex;

Model 2, additional adjustment for history of hypertension, duration of diabetes, smoking status, baseline HbA1c, postprandial plasma glucose, non-HDL cholesterol, eGFR, the presence of multivessel disease, peak value of troponin I and baseline LVEF;

Model 3, additional adjustment for medication use of oral hypoglycemic agents, insulin, beta blocker and ACEI/ARB;

**P* for trend. ACEI, angiotensin-converting enzyme inhibitor; ARB, angiotensin receptor blocker; CI, confidence interval; CV, coefficient of variation; eGFR, estimated glomerular filtration rate; FPG, fasting plasma glucose; HbA1c, glycated hemoglobin A1c; HDL, high-density lipoprotein; LVAR, left ventricular adverse remodeling; LVEF, left ventricular ejection fraction; OR, odds ratio; SD, standard deviation; STEMI, ST-segment elevation myocardial infarction; VIM, variability independent of the mean.
